# Retraction: Upregulated TRIM29 promotes proliferation and metastasis of nasopharyngeal carcinoma via PTEN/AKT/mTOR signal pathway

**DOI:** 10.18632/oncotarget.28889

**Published:** 2026-05-20

**Authors:** Xiao-Min Zhou, Rui Sun, Dong-Hua Luo, Jian Sun, Mei-Yin Zhang, Meng-He Wang, Yang Yang, Hui-Yun Wang, Shi-Juan Mai

**Affiliations:** ^1^State Key Laboratory of Oncology in South China, Sun Yat-Sen University Cancer Center, Guangzhou, China; ^2^Collaborative Innovation Center for Cancer Medicine, Sun Yat-Sen University Cancer Center, Guangzhou, China; ^3^Department of Nasopharyngeal Carcinoma, Sun Yat-Sen University Cancer Center, Guangzhou, China; ^*^These authors have contributed equally to this work

**This article has been retracted:** Following an investigation into this paper, several instances of data irregularity were discovered, including:

- Figure 3A: The GAPDH western blot duplicates the GAPDH image in Figure 5C. Additionally, the TRIM29 blot was reused in Figure 6A as E-Cadherin (flipped and reversed).
- Figures 5C and 6B: The TRIM29 western blot in Figure 5C and the Vimentin panel in Figure 6B are duplicates.
- Figure 5D: Overlap was identified between the transwell assay invasion panels for Si#1 and Si#2.
- Figures 6A and 7A: The Vimentin western blot in Figure 6A and the TRIM29 blot in Figure 7A are duplicates.
- Figure 7A: The western blots for SK6 and AKT are duplicates.
- Figure 8A, 8B: The 6-10B/vector panel in Figure 8A overlaps with the 6-10B/TRIM29 panel in Figure 8B.

The authors did not respond to requests for comment or provide the original data. Consequently, the editorial decision has been made to retract this paper. Oncotarget has reached out to all authors to notify them of this retraction but has received no response.

Original article: Oncotarget. 2016; 7:13634–13650. 13634-13650. https://doi.org/10.18632/oncotarget.7215

